# Finite element analysis of sagittal screw expander appliance in the treatment of anterior maxillary hypoplasia

**DOI:** 10.3389/fbioe.2023.1245764

**Published:** 2023-10-26

**Authors:** Jian Zhang, Caiyun Liu, Yan Dong

**Affiliations:** ^1^ College of Stomatology, Dalian Medical University, Dalian, Liaoning, China; ^2^ Affiliated Hospital of Stomatology, Dalian Medical University, Dalian, Liaoning, China

**Keywords:** finite element analysis, anterior maxillary hypoplasia, sagittal screw expander appliance, displacement, stress distribution

## Abstract

The skeletal anterior crossbite is a common malocclusion in clinic. However, there have been no reports on the maxillary sagittal expansion to correct the premaxillary hypoplasia, which greatly influences the facial morphology and masticatory function, using finite element analysis. In the present study, a three-dimensional finite element model of craniomaxillofacial complex with maxillary sagittal hypoplasia is constructed and the treatment for premaxillary hypoplasia by the sagittal screw expander appliance is simulated. The hypoplasia of the left premaxilla is more serious than that of the right and thus the size of the left part of premaxillary expander baseplate is designed to be larger than that of the right part and the loading is applied at 10° leftward to the sagittal plane and 30° forward and downward to the maxillary occlusal plane. The displacements or equivalent stress distributions of the maxilla, teeth and their periodontal ligaments, are analyzed under the loads of 5.0 N, 10.0 N, 15.0 N, and 20.0 N. Consequently, as the load increases, the displacements or equivalent stresses of the maxilla, teeth and their periodontal ligaments all increase. Almost the whole premaxilla markedly move forward, downward, and leftward while other areas in the craniomaxillofacial complex remain almost static or have little displacement. The equivalent stress concentration zone of the maxilla mainly occurs around and in front of the incisive foramina. The displacements of left premaxilla are generally greater than those of the right under the loading forces. The maximum equivalent stress on the teeth and their periodontal ligaments are 2.34E-02 MPa and 2.98E-03 MPa, respectively. Taken together, the sagittal screw expander appliance can effectively open the premaxillary suture to promote the growth of the premaxilla. An asymmetrical design of sagittal screw expander appliance achieves the asymmetric expansion of the premaxilla to correct the uneven hypoplasia and obtains the more symmetrical aesthetic presentation. This study might provide a solid basis and theoretical guidance for the clinical application of sagittal screw expander appliance in the efficient, accurate, and personalized treatment of premaxillary hypoplasia.

## Introduction

The skeletal anterior crossbite is a common malocclusion in clinic (Lombardo et al., 2020; De Ridder et al., 2022), which mainly induced by hypoplasia of the maxilla, macroplastia of the mandible, or both (Zhang et al., 2023). For sagittal maxillary hypoplasia, orthopedic force is generally applied by the protraction appliance and transmitted to the sutural tissues in the craniomaxillofacial complex such as zygomaticomaxillary, pterygopalatine, zygomaticotemporal and frontomaxillary sutures to effectively promote the sagittal growth of the maxilla and correct the malocclusion relationship between the upper and lower dental arches (Matsumoto and Tanna, 2021; Vracar et al., 2021; Kamath et al., 2022; Wang et al., 2022). However, this treatment only can promote the sagittal growth of the posterior maxilla, but not the anterior, which greatly influences the facial morphology and masticatory function.

The premaxilla, where the four maxillary incisors are (WOO, 1949; Barteczko and Jacob, 2004), is closely associated with the development of the human facial morphology and is delimited by a suture that goes from the incisive foramina to the region between the lateral incisors and canines (Lisson and Kjaer, 1997). This suture goes down from the junction of the maxillary and premaxillary growth centers, near the lower portion of the pyriform aperture, to the alveolar margin of the canines, crossing the palate to the incisive foramina. Trevizan et al. (2018) analyzed the premaxillary sutures in 1138 human dry skulls and found that all sutures gradually closed at a rate of 3.72% per year from birth to 12 years old and were not fully closed at the age of 12 yet. Therefore, during the peak and pre-peak growth periods of children, the premaxillary suture can be opened by expander appliance to promote the growth of premaxilla, which can correct the hypoplasia of the anterior maxilla effectively.

Up to now, finite element analyses on the treatment of maxillary hypoplasia have mainly focused on the protraction (Garg et al., 2023), transverse expansion of the maxilla (Fernandes et al., 2021), and the interaction between them (Balakrishnan et al., 2023). Although clinically, a sagittal screw expander appliance placed on the palate of the maxilla is used to open the premaxillary suture (Farronato et al., 2011; Maspero et al., 2020), there have been no reports on the analysis of sagittal screw expander appliance in the treatment for the anterior maxillary hypoplasia using the finite element method. The purpose of this study is to investigate the effects of sagittal screw expander appliance on the anterior maxillary hypoplasia by finite element analysis, providing the potent basis for the clinical treatment of premaxillary hypoplasia.

## Materials and methods

**FIGURE 1:**
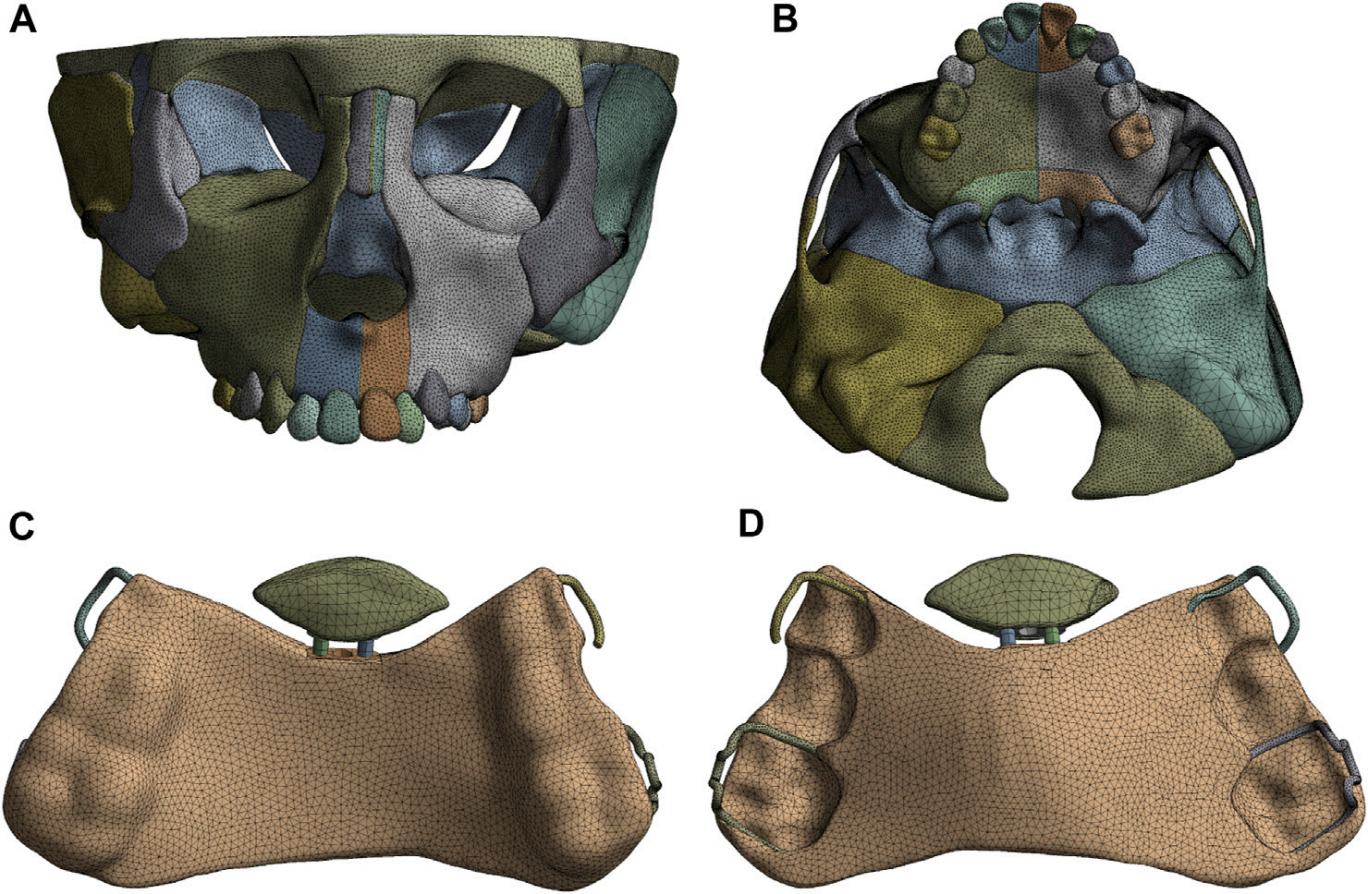
The finite element model of craniomaxillofacial complex **(A)**, Frontal view; **(B)**, Palatal view and sagittal screw expander appliance **(C)**, Lingual view; **(D)**, Palatal view.

The finite element model of the craniomaxillofacial complex, consisting of bones, sutures, teeth, and periodontal ligaments, is constructed from the Cone-Beam Computed Tomography (CBCT) images of a 9-year-old boy with skeletal anterior crossbite at the mixed dentition stage by using MIMICS 19.0 (Materialise, Leuven, Belgium), Geomagic Wrap 2017 (Geomagic Inc., Utah, United States of America), and Ansys 18.2 (ANSYS Inc., Canonsburg, PA) (Figures 1A, B). In the model, hypoplasia of the left anterior maxilla is more severe than that of the right. The width of the periodontal ligament and premaxillary suture is 0.2 mm (Kojima and Fukui, 2006), and the other maxillofacial sutures is 0.5 mm wide (Fricke-Zech et al., 2012).

**FIGURE 2:**
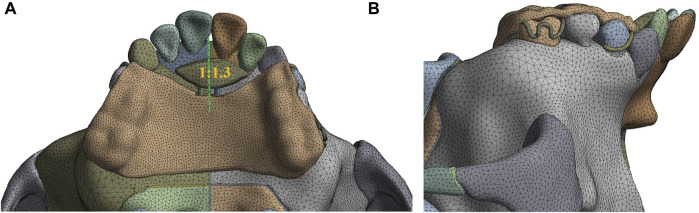
3D model assembled with maxillofacial complex and sagittal screw expander appliance **(A)** The area ratio of the right to the left part of anterior baseplate; **(B)** Buccal view of the appliance.

The computer aided design (CAD) model of the sagittal screw expander appliance with resin baseplate of an average thickness of 2.0 mm and stainless steel clasp of a diameter of 0.8 mm is established by using Solidworks 2020 (Dassault Systems, Concord, MA) (Figures 1C,D). The anterior baseplate is placed on the palate of the premaxilla and the area of the left part of anterior baseplate is 1.3 times larger than that of the right (Figure 2A). The posterior baseplate is connected to the occlusal splint, and to prevent premature loss of the maxillary second deciduous molars with obvious root resorption, the occlusal splint does not come into contact with them. The clasps are placed on the crowns of the first premolar and first molar of the maxilla as shown in Figure 2B.

The finite element model assembled from the craniomaxillofacial complex and sagittal screw expander appliance models consists of 1,002,520 elements ranging in size from 0.3 to 1.0 mm and 1,151,917 nodes. Furthermore, the materials involved are all assumed to be continuous, homogeneous, and isotropic linear elastic, with Young’s moduli and Poisson’s ratios shown in Table 1 (Weinstein et al., 1980; Serpe et al., 2014; Tanaka et al., 2015; Zarrati et al., 2015; Li et al., 2020; Duanmu et al., 2021; Sujaritwanid et al., 2021).

**TABLE 1:** The Young’s moduli and Poisson’s ratios for all materials.

Material	Young’s modulus (MPa)	Poisson’s ratios
Bone	10000	0.3
Suture	0.68	0.47
Tooth	18600	0.31
Periodontal ligament	68.9	0.45
Steel	200000	0.3
Baseplate	4500	0.35

**FIGURE 3:**
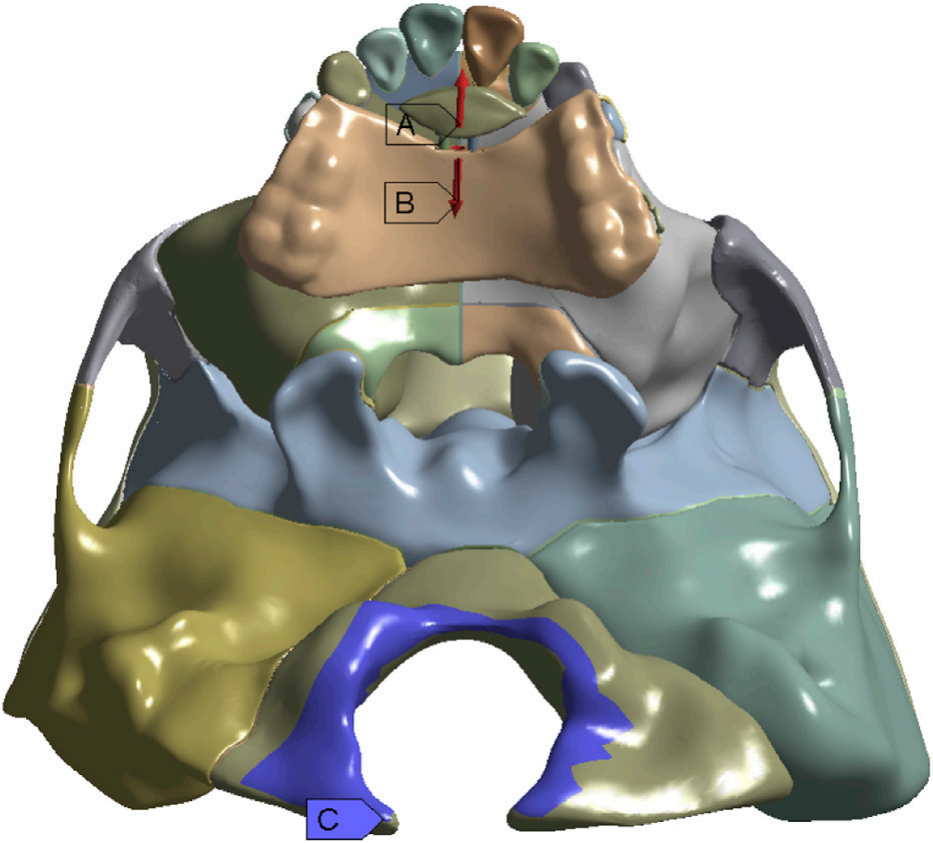
Loads **(A,B)** and fixed constraints **(C)** in the finite element model.

**FIGURE 4:**
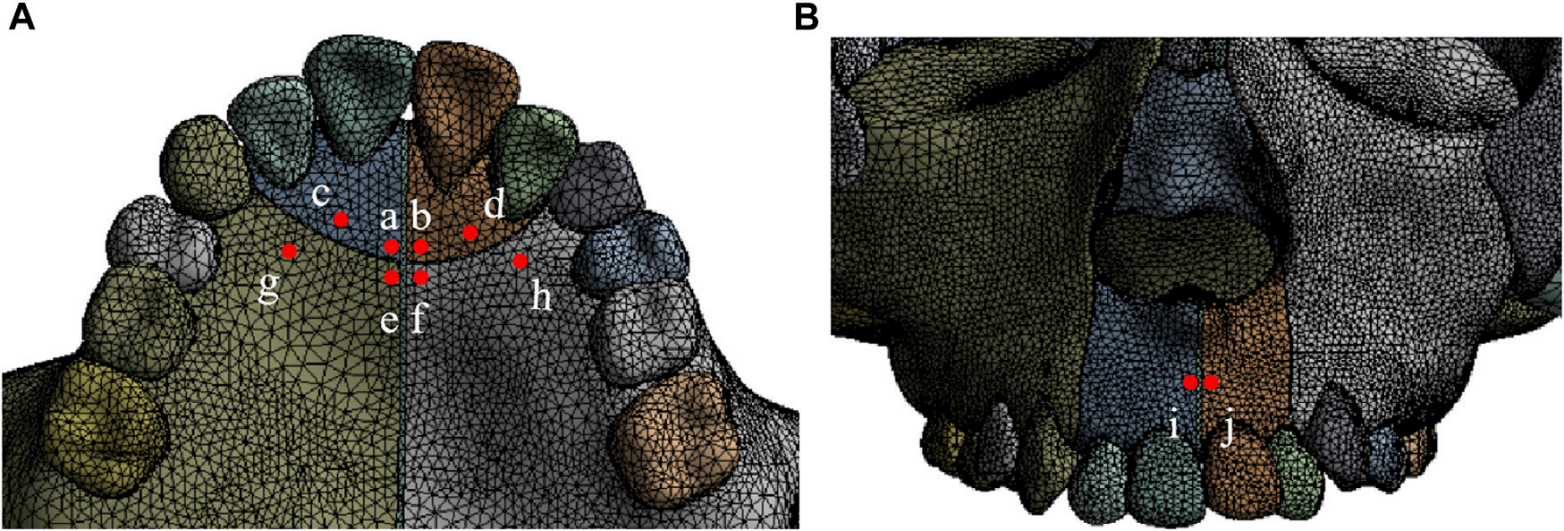
Ten landmarks on the anterior maxilla **(A)** ab, incisive foramen; cd, lateral incisor palatal point; ef, symmetrical point of incisive foramen; gh, canine palatal point; **(B)** ij, A.subspinale.

The three-dimensional coordinate system is defined by the occlusal plane as the *X*-axis (sagittal), *Y*-axis (vertical), and *Z*-axis (coronal). Positive values represent backward, upward, and leftward displacements on the X, Y, and *Z*-axis, respectively. The expansion screw is activated by loads of 5.0 N, 10.0 N, 15.0 N, and 20.0 N at 30° forward and downward to the maxillary occlusal plane and 10° leftward to the sagittal plane, and the foramen magnum is fixed (Figure 3) (Ge et al., 2012; Lee and Baek, 2012; Lee et al., 2016; Shyagali et al., 2023). As shown in Figure 4, ten landmarks include incisive foramen points (a,b), lateral incisor palatal points (c,d), symmetrical points of incisive foramina with the premaxillary suture as the symmetry axis (e,f), canine palatal points (g,h), and the A. subspinales (i,j).

By using Ansys 18.2 (ANSYS Inc., Canonsburg, PA), the displacements on the *X*, *Y*, *Z*-axis of ten landmarks and maxilla, the total displacement and equivalent stress distribution of the maxilla, and the equivalent stress distributions of the maxillary first premolars, first molars and their periodontal ligaments, are analyzed under the four loads of 5.0 N, 10.0 N, 15.0 N, and 20.0 N.

## Results

As the load increases from 5.0 N to 20.0 N, the absolute displacement values of all landmarks in the *X*-axis direction increase. The landmarks on the premaxilla (A.subspinales, incisive foramen points, and lateral incisor palatal points) move forward and the absolute displacement values of landmarks on the left premaxilla are all greater than those on the right. When the load is 20.0 N, the forward movement distances of the left/right incisive foramen point and lateral incisor palatal point, are 3.78E-04/3.48E-04 mm, 4.65E-04/3.85E-04 mm, respectively. On the maxilla region behind the premaxillary suture, the symmetrical points of incisive foramina and canine palatal points have little displacement (Table 2).

**TABLE 2:** Displacements of landmarks in the *X*-axis direction (mm).

Landmarks		Load			
		5.0 N	10.0 N	15.0 N	20.0 N
Incisive foramen point	Left	−9.46E-05	−1.89E-04	−2.84E-04	−3.78E-04
	Right	−8.70E-05	−1.74E-04	−2.61E-04	−3.48E-04
Lateral incisor palatal point	Left	−1.16E-04	−2.33E-04	−3.49E-04	−4.65E-04
	Right	−9.64E-05	−1.93E-04	−2.89E-04	−3.85E-04
A.subspinale	Left	−8.64E-06	−1.73E-05	−2.59E-05	−3.45E-05
	Right	−4.47E-07	−8.95E-07	−1.34E-06	−1.79E-06
Symmetrical point of incisive foramen	Left	1.39E-05	2.78E-05	4.17E-05	5.56E-05
	Right	1.93E-05	3.87E-05	5.80E-05	7.73E-05
Canine palatal point	Left	7.12E-06	1.42E-05	2.13E-05	2.85E-05
	Right	5.39E-06	1.08E-05	1.62E-05	2.15E-05

As the load increases, the absolute displacement values of ten landmarks in the *Y*-axis direction become larger, and all landmarks move downward except for the symmetrical points of incisive foramina. When the load is 20.0 N, the downward movement distances of the left/right incisive foramen point, lateral incisor palatal point, and A. subspinale are 4.94E-04/4.61E-04 mm, 4.01E-04/3.59E-04 mm, and 1.26E-04/1.15E-04 mm, respectively. The symmetrical points of incisive foramina and canine palatal points behind the premaxillary suture remain almost static (Table 3).

**TABLE 3:** Displacements of landmarks in the *Y*-axis direction (mm).

Landmarks		Load			
		5.0 N	10.0 N	15.0 N	20.0 N
Incisive foramen point	Left	−1.23E-04	−2.47E-04	−3.70E-04	−4.94E-04
	Right	−1.15E-04	−2.30E-04	−3.45E-04	−4.61E-04
Lateral incisor palatal point	Left	−1.00E-04	−2.00E-04	−3.00E-04	−4.01E-04
	Right	−8.97E-05	−1.79E-04	−2.69E-04	−3.59E-04
A.subspinale	Left	−3.14E-05	−6.29E-05	−9.43E-05	−1.26E-04
	Right	−2.89E-05	−5.77E-05	−8.66E-05	−1.15E-04
Symmetrical point of incisive foramen	Left	4.61E-07	9.23E-07	1.38E-06	1.85E-06
	Right	1.16E-06	2.32E-06	3.48E-06	4.64E-06
Canine palatal point	Left	−9.87E-08	−1.97E-07	−2.96E-07	−3.95E-07
	Right	−1.59E-07	−3.17E-07	−4.76E-07	−6.35E-07

As the load increases, in the *Z*-axis direction, displacements of ten landmarks increase. The displacements of landmarks on the left premaxilla are significantly greater than those of the corresponding landmarks on the right except for the A. subspinales. When the load is 20.0 N, displacements of the left incisive foramen point and lateral incisor palatal point are 1.47E-04 mm and 1.41E-04 mm, respectively, while the right corresponding ones, A. subspinales, symmetrical points of incisive foramina and canine palatal points all have little displacement (Table 4).

**TABLE 4:** Displacements of landmarks in the *Z*-axis direction (mm).

Landmarks		Load			
		5.0 N	10.0 N	15.0 N	20.0 N
Incisive foramen point	Left	3.68E-05	7.37E-05	1.10E-04	1.47E-04
	Right	1.05E-05	2.09E-05	3.14E-05	4.18E-05
Lateral incisor palatal point	Left	3.52E-05	7.04E-05	1.06E-04	1.41E-04
	Right	7.19E-06	1.44E-05	2.16E-05	2.88E-05
A.subspinale	Left	2.41E-06	4.82E-06	7.24E-06	9.65E-06
	Right	9.61E-06	1.92E-05	2.88E-05	3.85E-05
Symmetrical point of incisive foramen	Left	2.42E-06	4.85E-06	7.27E-06	9.70E-06
	Right	5.04E-06	1.01E-05	1.51E-05	2.02E-05
Canine palatal point	Left	2.10E-06	4.20E-06	6.30E-06	8.40E-06
	Right	9.25E-06	1.85E-05	2.77E-05	3.70E-05

**FIGURE 5:**
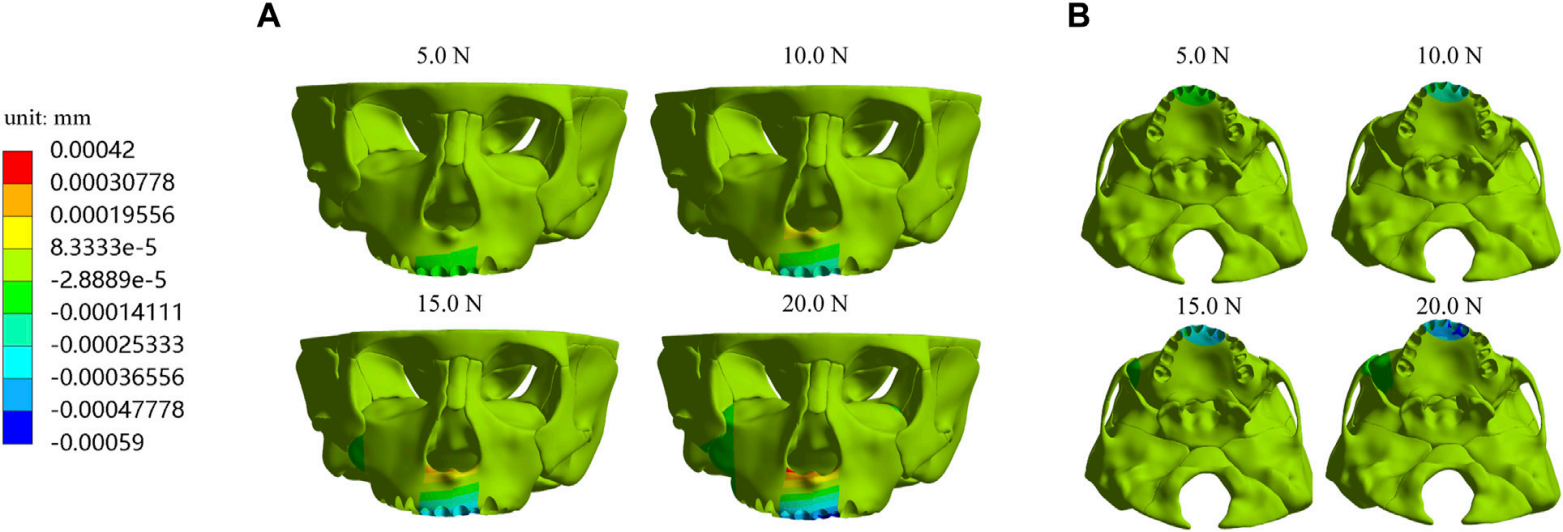
Displacement of maxilla in the *X*-axis direction **(A)** Frontal view; **(B)** Palatal view.

**FIGURE 6:**
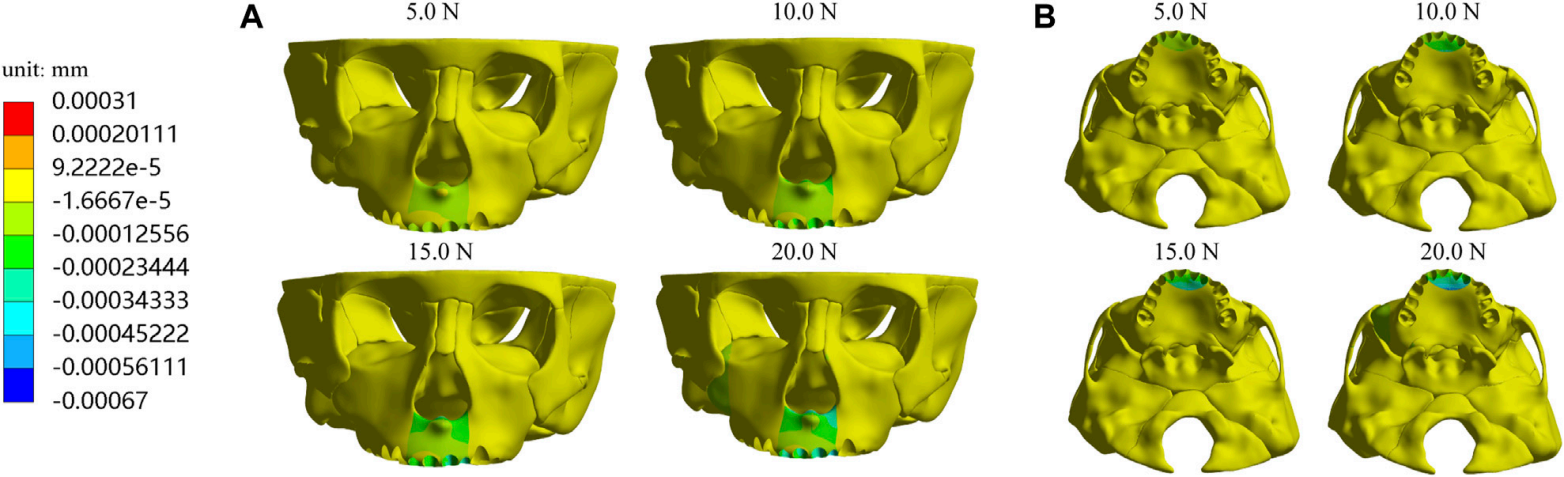
Displacement of maxilla in the *Y*-axis direction **(A)** Frontal view; **(B)** Palatal view.

**FIGURE 7:**
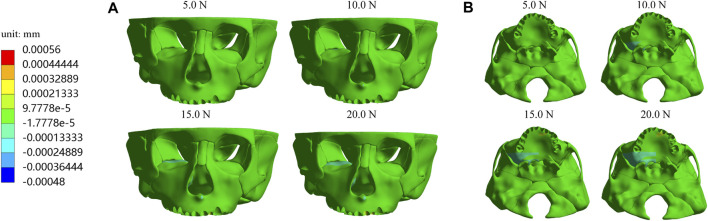
Displacement of maxilla in the *Z*-axis direction **(A)** Frontal view; **(B)** Palatal view.

**FIGURE 8:**
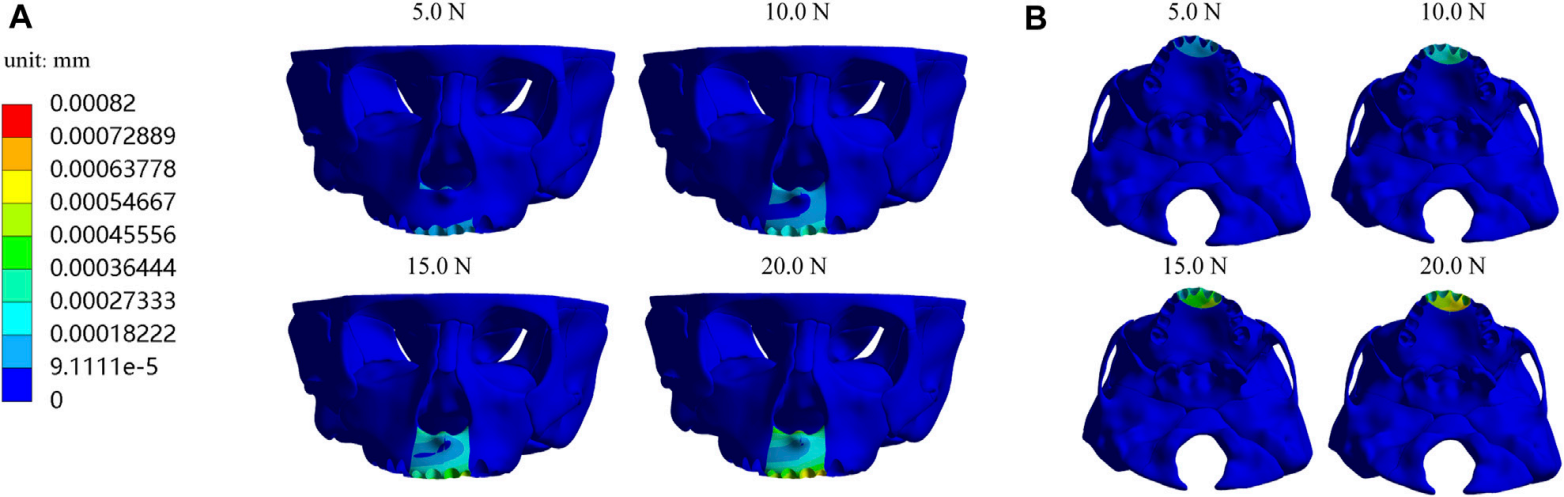
Total displacement of maxilla **(A)** Frontal view; **(B)** Palatal view.

Furthermore, we investigate displacements in the X, Y, and *Z*-axis directions and the total displacement of the maxilla, which all increase as the load increases. In the *X*-axis direction, most of the premaxilla moves forward and the greatest displacement, 5.61E-04 mm, occurs in the palatal alveolar process of left lateral incisor (Figure 5). In the *Y*-axis direction, as shown in Figure 6, most of the premaxilla moves downward and the largest displacement appears around the incisive foramina. On the *Z*-axis, the left premaxilla has a remarkable leftward movement (Figure 7), and the maximum movement distance, 1.74E-04 mm, occurs in the palatal alveolar process of the left lateral incisor, while the right premaxilla has little displacement. Figure 8 shows that under the loading forces, most of the premaxilla has obvious displacement while the maxillary region behind the premaxillary suture almost has no displacement, and the total displacements of the left premaxilla are generally greater than those of the right.

**FIGURE 9:**
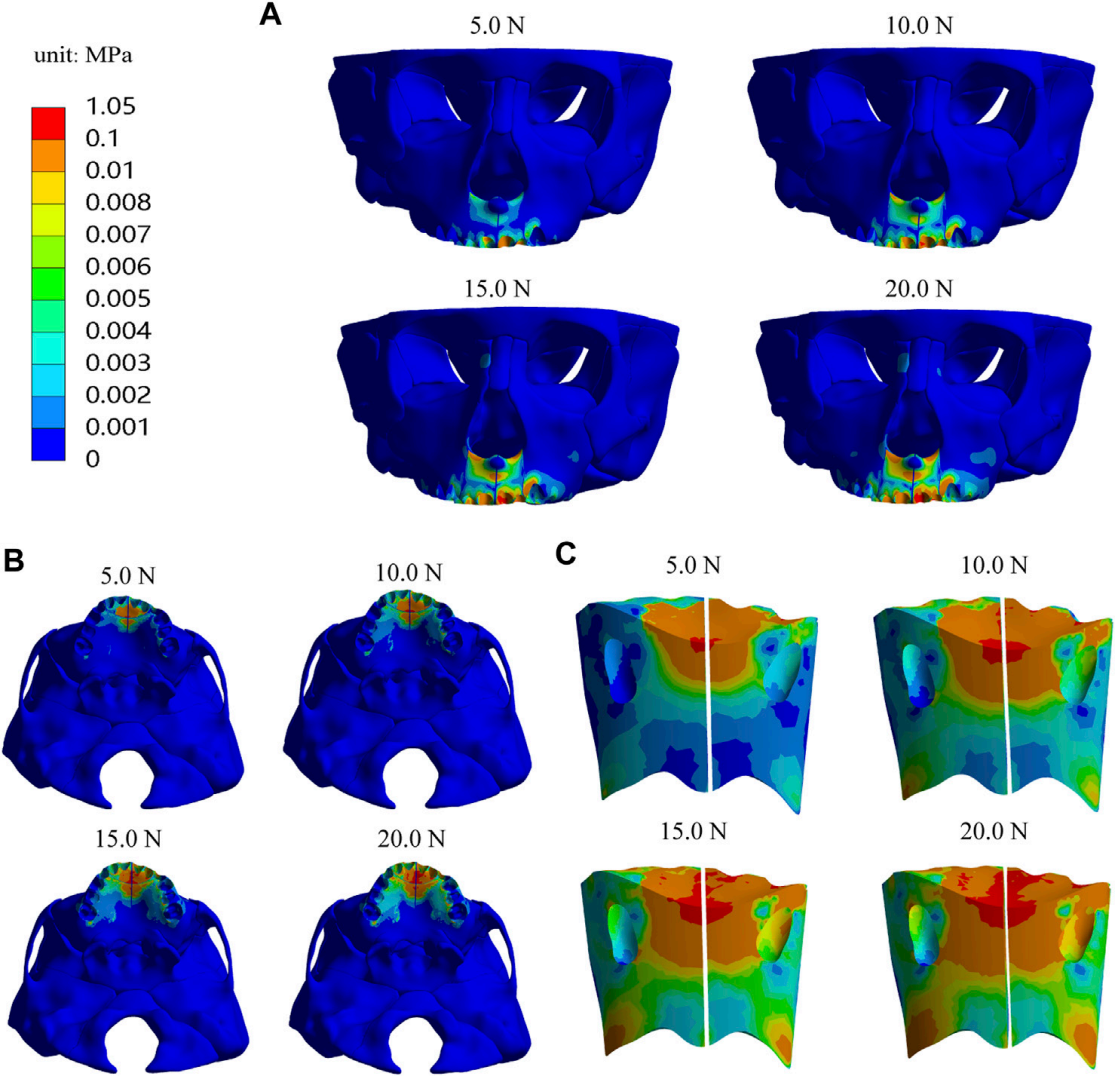
The equivalent stress distribution of maxilla **(A)** Frontal view; **(B)** Palatal view; **(C)** Profile view of the premaxillary suture.

**FIGURE 10:**
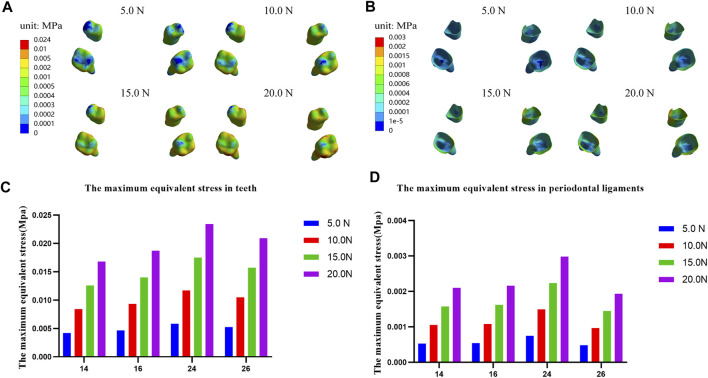
Equivalent stress distribution and the maximum equivalent stress in teeth **(A,C)** and their periodontal ligaments **(B,D)**.

In addition, we analyze the equivalent stress on the maxilla, the first premolar, first molar and their periodontal ligaments under the four loads and the results showed that the equivalent stresses are enhanced when the load increases. The stress distributions on the maxilla and the profile of the premaxillary suture are shown in Figure 9, and it can be observed that the stress concentrations of the maxilla mainly occur in a large area around and in front of the incisor foramina. The largest stress concentration area of the teeth is located in the mesial region of the left first premolar crown (Figure 10A) and that of the periodontal ligament appears in the palatal cervical region of the left first premolar periodontal ligament (Figure 10B). When the load is 20.0 N, the maximum equivalent stress are 2.34 E−02 and 2.98 E−03 MPa on the left maxillary first premolar and its periodontal ligament, respectively (Figures 10C,D).

## Discussion

Nowadays, finite element analysis has been widely used in researches on the treatment of the different types of malocclusion. It can effectively simulate the orthodontics process and predict the treatment effects (Mitani et al., 2018; Choi et al., 2021; Maheshwari et al., 2023; Somaskandhan et al., 2023). However, there have been no reports on the analysis of maxillary sagittal expansion to correct the premaxilla hypoplasia using finite element method.

In the present study, three-dimensional finite element models of the craniomaxillofacial complex and sagittal screw expander appliance are constructed and then the treatment of premaxillary hypoplasia is simulated. It is worth noting that the hypoplasia of the left premaxilla is more serious than that of the right, showing an asymmetric premaxillary hypoplasia. Some studies have indicated that an asymmetric orthodontic appliance can effectively correct the asymmetry. Choi et al. (2021) employed a palatal asymmetric transverse expansion appliance and achieved differential displacements between the left and right maxilla using finite element analysis. Furthermore, in a clinical treatment for a 14-year-old girl with more severe hypoplasia in her left maxilla compared to the right, they used an asymmetric transverse expansion appliance with the larger left baseplate section. The clinical treatment results confirmed that when asymmetric maxillary needs to be corrected, asymmetric maxillary expansion could produce more symmetric orthodontic outcomes. Chen et al. (2015) used finite element analysis to apply asymmetrical loads in the treatment of a patient with asymmetrical maxillary hypoplasia induced by the unilateral cleft lip and palate. The results demonstrated that the asymmetric loading induced asymmetrical displacements of the left and right maxilla, achieving a more symmetrical appearance. Sadeghi et al. (2019) suggested that employing an asymmetric loading was effective in generating asymmetric distal movement of the molars.

In the present study, the hypoplasia of the left premaxilla is more severe than that of the right, so we designed the anterior baseplate to be asymmetric, with the left part of premaxillary expander baseplate being larger than the right part, and applied the loading forces at 10° leftward to the sagittal plane. Simulation results indicate that an asymmetric appliance can effectively generate a larger displacement and growth in the left maxilla. Under different magnitudes of orthopedic forces, the forward, leftward, and downward displacements of the left premaxillary landmark b and d are all greater than those of the corresponding right landmark a and c, respectively (Table 2–4), and the total displacements of the left premaxilla are generally greater than those of the right part (Figure 8). These results suggest that the asymmetric design of the maxillary expander is beneficial for correcting the asymmetric hypoplasia of the premaxilla.

As the load increases from 5.0 N to 20.0 N, the absolute displacement values of all landmarks in the X, Y, and *Z*-axis directions increase (Table 2–4). In the *X*-axis direction, the landmarks (a, b, c, d) on the premaxilla all move forward, and their displacements reached the maximum under 20.0 N load, which are 3.48E-04, 3.78E-04, 3.85E-04, 4.65E-04 mm, respectively, while landmarks (e, f, g, h) located behind the premaxillary suture have little displacements. In the *Y*-axis direction, Point a, b, c, d, i, and j on the premaxilla all move downward significantly, and have the largest displacements under 20.0 N load, which are 4.61E-04, 4.94E-04, 3.59E-04, 4.01E-04, 1.15E-04, and 1.26E-04 mm, respectively, while Point e, f, g and h behind the premaxillary suture almost have no displacements. Taken together, it can be concluded that the loading forces applied in the direction with 30° forward and downward to the occlusal plane can cause the premaxilla to move forward and downward on the whole. Therefore, the sagittal screw expander appliance can open the premaxillary suture and hence effectively promote the growth of the premaxilla. In addition, A. subspinales have little forward displacements in the *X*-axis direction, so we plan to use the sagittal screw expander appliance in combination with the protraction to achieve further forward displacement of the whole maxilla in further research.

In the *Z*-axis direction, all the landmarkers move left, which is due to the left part of the baseplate on the premaxilla being significantly larger than the right part and loading forces applied at 10° leftward to the sagittal plane. The results show that the maximum displacement values of landmark b and d on the left premaxilla are 1.47E-04 and 1.41E-04 mm, respectively, while the right premaxilla has remain almost static, which suggest that the leftward development of the left premaxilla with the more severe hypoplasia is promoted effectively.

In addition, the equivalent stress distribution and displacement of the maxilla are analyzed and from the stress distribution on the maxilla and the profile of the premaxillary suture displayed in Figure 9, the equivalent stress concentration zone is mainly located in a large area around and in front of the incisive foramina, which indicates that orthopedic forces applied by the sagittal screw expander appliance are transmitted effectively to the premaxilla, contributing to the expansion of premaxillary suture. Interestingly, by the displacement analysis, we find that the premaxilla has obvious displacement and the maximum forward and leftward movement distances, 5.61E-04 and 1.74E-04 mm, respectively, appear in the region around the palatal alveolar process of the left lateral incisor with severe hypoplasia, suggesting that the premaxilla is accurately expanded by personalized design of sagittal screw expander appliance (Figures 5, 7, 8).

Moreover, we studied the equivalent stress distribution on the maxillary first premolars and first molars with the clasp and occlusal splint, and their periodontal ligaments. The results show that the equivalent stress increase with the increase of load. The maximum value on the teeth and the periodontal ligaments are 2.34E-02 MPa and 2.98E-03 MPa, respectively, on the left maxillary first premolar and its periodontal ligaments under 20.0 N load (Figure 10), which are far lower than the yield strength of the tooth (97.8 MPa) (Staninec et al., 2002) and the periodontal ligament (0.026 MPa) (Lee, 1965). Therefore, when the sagittal screw expander appliance is used to promote the growth of the premaxilla, it will not cause the tooth fracture and periodontal ligament injury.

## Conclusion

The three-dimensional finite element models of craniomaxillofacial complex with sagittal maxillary hypoplasia and the sagittal screw expander appliance are successfully constructed and the treatment of the anterior maxillary hypoplasia by the sagittal screw expander appliance is simulated using CAD softwares. Under the orthopedic force ranged from 5.0 to 20.0 N, the screw expander appliance can open the premaxillary suture without causing the tooth fracture and periodontal ligament injury, and the greater the applied orthopedic force, the greater the opening of the premaxillary suture, which has suggested that sagittal expansion can effectively promote the growth of the premaxilla. Moreover, for the asymmetric anterior maxillary hypoplasia, we design an asymmetric appliance and achieve asymmetric expansion of the premaxilla to correct the uneven hypoplasia and obtain the more symmetrical aesthetic presentation. Taken together, this study might provide a solid basis and theoretical guidance for the clinical application of sagittal screw expander appliance in the efficient, accurate, and personalized treatment of premaxillary hypoplasia.

## Data Availability

The original contributions presented in the study are included in the article/supplementary material, further inquiries can be directed to the corresponding author.

## References

[B1] Balakrishnan R. Sengottuvel N. Altaf S. K. Bhandari P. K. Kumaragurubaran P. Antony M. (2023). Three-dimensional finite element analysis of maxillary protraction using diverse modes of rapid palatal expansion. Cureus 15 (3), e36328. 10.7759/cureus.36328 37077604 PMC10108977

[B2] Barteczko K. Jacob M. (2004). A re-evaluation of the premaxillary bone in humans. Anat. Embryol. Berl. 207 (6), 417–437. 10.1007/s00429-003-0366-x 14760532

[B3] Chen Z. Pan X. Zhao N. Chen Z. Shen G. (2015). Asymmetric maxillary protraction for unilateral cleft lip and palate patients using finite element analysis. J. Craniofac Surg. 26 (2), 388–392. 10.1097/SCS.0000000000001337 25759916

[B4] Choi J. Y. Choo H. Oh S. H. Park J. H. Chung K. R. Kim S. H. (2021). Finite element analysis of C-expanders with different vertical vectors of anchor screws. Am. J. Orthod. Dentofac. Orthop. 159 (6), 799–807. 10.1016/j.ajodo.2020.02.024 33762139

[B5] De Ridder L. Aleksieva A. Willems G. Declerck D. Cadenas de Llano-Pérula M. (2022). Prevalence of orthodontic malocclusions in healthy children and adolescents: a systematic review. Int. J. Environ. Res. Public Health. 19 (12), 7446. 10.3390/ijerph19127446 35742703 PMC9223594

[B6] Duanmu Z. Liu L. Deng Q. Ren Y. Wang M. (2021). Development of a biomechanical model for dynamic occlusal stress analysis. Int. J. Oral Sci. 13 (1), 29. 10.1038/s41368-021-00133-5 34493701 PMC8423745

[B7] Farronato G. Maspero C. Esposito L. Briguglio E. Farronato D. Giannini L. (2011). Rapid maxillary expansion in growing patients. Hyrax versus transverse sagittal maxillary expander: a cephalometric investigation. Eur. J. Orthod. 33 (2), 185–189. 10.1093/ejo/cjq051 21059876

[B8] Fernandes L. C. Farinazzo Vitral R. W. Noritomi P. Y. Maximiano G. S. José da Silva Campos M. (2021). Influence of the hyrax expander screw position on displacement and stress distribution in teeth: a study with finite elements. Am. J. Orthod. Dentofac. Orthop. 160 (2), 266–275. 10.1016/j.ajodo.2020.04.031 34006424

[B9] Fricke-Zech S. Gruber R. M. Dullin C. Zapf A. Kramer F. J. Kubein-Meesenburg D. (2012). Measurement of the midpalatal suture width. Angle Orthod. 82 (1), 145–150. 10.2319/040311-238.1 21812573 PMC8881041

[B10] Garg D. Rai P. Tripathi T. Kanase A. (2023).Effects of different force directions of intra-oral skeletally anchored maxillary protraction on craniomaxillofacial complex, in Class III malocclusion: a 3D finite element analysis, Dent. Press J. Orthod., 5e2220377, 27. 10.1590/2177-6709.27.5.e2220377.oar PMC9829108 36629626

[B11] Ge Y. S. Liu J. Chen L. Han J. L. Guo X. (2012). Dentofacial effects of two facemask therapies for maxillary protraction. Angle Orthod. 82(6):1083–1091. 10.2319/012912-76.1 22639823 PMC8813143

[B12] Kamath A. Sudhakar S. S. Kannan G. Rai K. Sb A. (2022). Bone-anchored maxillary protraction (BAMP): a review. J. Orthod. Sci. 11, 8. 10.4103/jos.jos_153_21 35754417 PMC9214452

[B13] Kojima Y. Fukui H. (2006). A numerical simulation of tooth movement by wire bending. Am. J. Orthod. Dentofac. Orthop. 130 (4), 452–459. 10.1016/j.ajodo.2005.01.028 17045144

[B14] Lee B. W. (1965). Relationship between tooth-movement rate and estimated pressure applied. J. Dent. Res. 44 (5), 1053. 10.1177/00220345650440051001 5213010

[B15] Lee H. Nguyen A. Hong C. Hoang P. Pham J. Ting K. (2016). Biomechanical effects of maxillary expansion on a patient with cleft palate: a finite element analysis. Am. J. Orthod. Dentofac. Orthop. 150 (2), 313–323. 10.1016/j.ajodo.2015.12.029 PMC5557378 27476365

[B16] Lee N. K. Baek S. H. (2012). Stress and displacement between maxillary protraction with miniplates placed at the infrazygomatic crest and the lateral nasal wall: a 3-dimensional finite element analysis. Am. J. Orthod. Dentofac. Orthop. 141 (3), 345–351. 10.1016/j.ajodo.2011.07.021 22381495

[B17] Li X. Kang T. Zhan D. Xie J. Guo L. (2020). Biomechanical behavior of endocrowns vs fiber post-core-crown vs cast post-core-crown for the restoration of maxillary central incisors with 1 mm and 2 mm ferrule height: a 3D static linear finite element analysis. Med. Baltim. 99 (43), e22648. 10.1097/MD.0000000000022648 PMC7581096 33120754

[B18] Lisson J. A. Kjaer I. (1997). Location of alveolar clefts relative to the incisive fissure. Cleft Palate Craniofac J. 34 (4), 292–296. 10.1597/1545-1569_1997_034_0292_loacrt_2.3.co_2 9257019

[B19] Lombardo G. Vena F. Negri P. Pagano S. Barilotti C. Paglia L. (2020). Worldwide prevalence of malocclusion in the different stages of dentition: a systematic review and meta-analysis. Eur. J. Paediatr. Dent. 21 (2), 115–122. 10.23804/ejpd.2020.21.02.05 32567942

[B20] Maheshwari A. Chawda D. N. Kushwah A. Agarwal R. K. Golwara A. K. Dixit P. B. (2023).Comparative evaluation of displacement and stress distribution pattern during mandibular arch distalization with extra and inter-radicular mini-implants: a three-dimensional finite element study, Dent. Press J. Orthod., e2321373, 28. 10.1590/2177-6709.28.2.e2321373.oar PMC10229115 37255133

[B21] Maspero C. Cavagnetto D. Fama A. Giannini L. Galbiati G. Farronato M. (2020). Hyrax versus transverse sagittal maxillary expander: an assessment of arch changes on dental casts. A retrospective study. Saudi Dent. J. 32 (2), 93–100. 10.1016/j.sdentj.2019.06.003 32071538 PMC7016244

[B22] Matsumoto K. Tanna N. (2021).Maxillary protraction and vertical control utilizing skeletal anchorage for midfacial-maxillary deficiency, Dent. Press J. Orthod., e2120114, 26. 10.1590/2177-6709.26.6.e2120114.oar PMC8690863 34932708

[B23] Mitani Y. Choi B. Choi J. (2018). Anterosuperior protraction of maxillae using the extraoral device, RAMPA; finite element method. Comput. Methods Biomech. Biomed. Engin 21 (13), 722–729. 10.1080/10255842.2018.1514498 30369258

[B24] Sadeghi S. Hedayati Z. Mousavi-Fard B. (2019). Comparison of two asymmetric headgear force systems: a finite element analysis. Dent. Press J. Orthod. 24 (2), 41.e1–41.e6. 10.1590/2177-6709.24.2.41.e1-6.onl PMC6526760 31116285

[B25] Serpe L. C. T. Torres L. A. G. de Freitas Pinto R. U. Toyofuku A. C. M. M. de Las Casas E. B. (2014). Maxillary biomechanical study during rapid expansion treatment with simplified model. J. Med. Imaging Health Inf. 4 (1), 137–141. 10.1166/jmihi.2014.1233

[B26] Shyagali T. R. Patidar R. Gupta A. Kapoor S. Tiwari A. (2023). Evaluation of stresses and displacement in the craniofacial region as a reaction to bone-anchored maxillary protraction in conjugation with posterior bite plane and rapid maxillary expansion in patients with Class III malocclusion: a finite element analysis study. Am. J. Orthod. Dentofac. Orthop. 164 (2), 253–264. 10.1016/j.ajodo.2022.12.015 36959013

[B27] Somaskandhan A. Kumar N. M. V. Vijayalakshmi R. D. (2023). Stress distribution and displacement in the maxillofacial complex during intrusion and distalization of the maxillary arch using miniplates versus mini-implants: a 3-dimensional finite element study. Prog. Orthod. 24 (1), 8. 10.1186/s40510-023-00455-6 36854939 PMC9975133

[B28] Staninec M. Marshall G. W. Hilton J. F. Pashley D. H. Gansky S. A. Marshall S. J. (2002). Ultimate tensile strength of dentin: evidence for a damage mechanics approach to dentin failure. J. Biomed. Mater. Res. 63 (3), 342–345. 10.1002/jbm.10230 12115767

[B29] Sujaritwanid K. Suzuki B. Suzuki E. Y. (2021). Comparison of one versus two maxillary molars distalization with iPanda: a finite element analysis. Prog. Orthod. 22 (1), 12. 10.1186/s40510-021-00356-6 33937947 PMC8089070

[B30] Tanaka O. M. Araújo E. A. Oliver D. R. Behrents R. G. (2015). A finite element analysis of the maxillary first molar PDL with maxillary protraction in a mixed dentition Class III malocclusion. Orthod. Craniofac Res. 18 (4), 242–250. 10.1111/ocr.12102 26333535

[B31] Trevizan M. Nelson Filho P. Franzolin S. O. B. Consolaro A. (2018). Premaxilla: up to which age it remains separated from the maxilla by a suture, how often it occurs in children and adults, and possible clinical and therapeutic implications: study of 1,138 human skulls. Dent. Press J. Orthod. 23 (6), 16–29. 10.1590/2177-6709.23.6.016-029.oin PMC6340201 30672982

[B32] Vracar T. R. Claro W. Vracar M. E. 2nd Jenkins R. S. Bland L. Dayeh A. A. (2021). Sutural deformation during bone-anchored maxillary protraction. J. Oral Biol. Craniofac Res. 11 (3), 447–450. 10.1016/j.jobcr.2021.05.008 34094844 PMC8167158

[B33] Wang J. Yang Y. Wang Y. Zhang L. Ji W. Hong Z. (2022). Clinical effectiveness of different types of bone-anchored maxillary protraction devices for skeletal Class III malocclusion: systematic review and network meta-analysis. Korean J. Orthod. 52 (5), 313–323. 10.4041/kjod21.264 35844098 PMC9512627

[B34] Weinstein A. M. Klawitter J. J. Cook S. D. (1980). Implant-bone interface characteristics of bioglass dental implants. J. Biomed. Mater. Res. 14 (1), 23–29. 10.1002/jbm.820140104 6987233

[B35] Woo J. K. (1949). Ossification and growth of the human maxilla, premaxilla and palate bone. Anat. Rec. 105 (4), 737–761. 10.1002/ar.1091050408 15409816

[B36] Zarrati S. Bahrami M. Heidari F. Kashani J. (2015). Three dimensional finite element analysis of distal abutment stresses of removable partial dentures with different retainer designs. J. Dent. (Tehran) 12 (6), 389–397. 26884772 PMC4754564

[B37] Zhang J. N. Lu H. P. Hou J. Wang Q. Yu F. Y. Zhong C. (2023). Deep learning-based prediction of mandibular growth trend in children with anterior crossbite using cephalometric radiographs. BMC Oral Health 23 (1), 28. 10.1186/s12903-023-02734-4 36650491 PMC9843828

